# Solution‐Processed Heterojunction Photodiodes Based on WSe_2_ Nanosheet Networks

**DOI:** 10.1002/smll.202304735

**Published:** 2023-09-21

**Authors:** Shixin Liu, Tian Carey, Jose Munuera, Kevin Synnatschke, Harneet Kaur, Emmet Coleman, Luke Doolan, Jonathan N. Coleman

**Affiliations:** ^1^ School of Physics CRANN & AMBER Research Centres Trinity College Dublin 2 Ireland; ^2^ Department of Physics Faculty of Sciences University of Oviedo C/Leopoldo Calvo Sotelo 18 Oviedo Asturias 33007 Spain

**Keywords:** 2D materials, photodetectors, solution processed heterojunctions, transition metal dichalcogenides

## Abstract

Solution‐processed photodetectors incorporating liquid‐phase‐exfoliated transition metal dichalcogenide nanosheets are widely reported. However, previous studies mainly focus on the fabrication of photoconductors, rather than photodiodes which tend to be based on heterojunctions and are harder to fabricate. Especially, there are rare reports on introducing commonly used transport layers into heterojunctions based on nanosheet networks. In this study, a reliable solution‐processing method is reported to fabricate heterojunction diodes with tungsten selenide (WSe_2_) nanosheets as the optical absorbing material and PEDOT: PSS and ZnO as injection/transport‐layer materials. By varying the transport layer combinations, the obtained heterojunctions show rectification ratios of up to ≈10^4^ at ±1 V in the dark, without relying on heavily doped silicon substrates. Upon illumination, the heterojunction can be operated in both photoconductor and photodiode modes and displays self‐powered behaviors at zero bias.

## Graphical abstract

Solution‐processed heterojunctions based on WSe_2_ nanosheet networks are fabricated. The obtained heterojunctions display a high rectification ratio of up to ≈10^4^ at ±1 V in the dark and can be used as photodetectors in both photoconductor (1 V) and photodiode (0 and −1 V) modes under illumination.

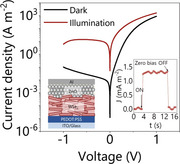

## 1 Introduction

Two‐dimensional (2D) transition metal dichalcogenides (TMDs) are a class of attractive materials for optoelectronic devices,^[^ ^1^ ^]^ due to their various and tunable bandgaps,^[^ ^2^ ^]^ high absorption coefficients,^[^ ^3^ ^]^ and strong light‐matter interactions,^[^ ^4^ ^]^ etc. It is expected that these materials could be used as active layers in ultra‐thin and light‐weight optoelectronic devices. Progress on such TMDs‐based devices was focused on mechanically exfoliated and chemical vapor‐deposited nanosheets, which are limited by scalability.^[^ ^5^ ^]^

However, alongside such traditional optoelectronic systems, the field of printed optoelectronics has been growing rapidly. In this area, the ability to fabricate low‐cost devices over large areas by solution processing is more important than achieving extremely high performance. Although organic materials long dominated printed optoelectronics, the last decade has demonstrated several promising devices incorporating liquid‐processed 2D materials, including solution‐processed 2D photodetectors.^[^ ^6^ ^]^

In order to fabricate 2D‐based electronic devices by solution processing or printing, one must first have access to printable inks, i.e., suspensions of 2D nanosheets in appropriate liquids. One way to produce such inks is using a technique known as liquid phase exfoliation (LPE). This method can be used to exfoliate layered materials in appropriate solvent media by either ultra‐sonication or high‐sheer mixing.^[^ ^7^ ^]^ It is facile and low‐cost and is so far the most efficient method to prepare various kinds of nanosheets in large quantities.^[^ ^8^ ^]^ Subsequent processing steps, involving centrifugation, solvent exchange, or addition of rheology modifiers are often used to convert the suspension into an ink. Such inks can be printed using common deposition techniques, such as spray coating,^[^ ^9^ ^]^ ink‐jet printing,^[^ ^10^ ^]^ and aerosol‐jet printing.^11^ ^]^ These processes have enabled a range of studies on solution‐processed optoelectronics using LPE TMDs nanosheet networks.^[^ ^6c,d^ ^]^

One important optoelectronic device is the photodetector, which is a device that responds to illumination via a change in its electrical properties. There are two types of solid‐state photodetectors: photoconductors and photodiodes. The device structure of a photoconductor is usually metal–semiconductor–metal fabricated either laterally or vertically.^[^ ^12^ ^]^ Its operation mechanism is based on the changing of the conductivity of the semiconductor via the photo generation of carriers, although the metal–semiconductor contact effects can also be likely involved. An external bias must be applied to the device to operate. The simplicity of the device structure has made this the most common LPE TMDs‐based photodetector.^[^ ^12^, ^13^ ^]^

Another type of photodetector is the photodiode which is based on carrier separation at a homo‐ or hetero‐junction interface (e.g., a pn junction). One advantage of photodiodes is that they can operate with or without an external bias, with the latter case allowing them to be self‐powered.^[^ ^14^ ^]^ Moreover, they can exhibit low dark current^[^ ^15^ ^]^ and fast photo responses,^[^ ^16^ ^]^ making photodiodes more promising than photodetectors despite their added structural complexity.

Recent research on solution‐processed TMD heterojunctions has focused on LPE TMDs deposited on heavily doped silicon to form heterojunctions.^[^ ^17^ ^]^ Given the high optoelectronic efficiency of silicon itself, it is still unclear of the importance of TMDs as the light‐absorbing layer in such heterojunction photodiodes. In contrast, there have been relatively few reports of TMDs‐based photodiodes where multiple layers in the heterojunction have been solution‐processed,^[^ ^18^ ^]^ especially using common transport layer materials. Transport layers are widely adopted in the field of solution‐processed optoelectronic devices to manipulate electronic properties of the interface, but not yet attempted and investigated for nanosheet networks.

One reason is the inherent difficulty in fabricating solution‐processed heterojunctions based on nanosheet networks, which has been largely underestimated.^[^ ^19^ ^]^ Due to the rigidity of nanosheets produced by LPE, they stack randomly on each other during solution processing (e.g., spray coating) resulting in the formation of a porous network (porosity ≈50%).^[^ ^19^, ^20^ ^]^ This allows any subsequently evaporated metal to diffuse into pores potentially forming electrical shorts. While this issue could be solved by increasing network thickness,^[^ ^9^, ^19^ ^]^ eventually the devices will become bulk‐ rather than interface‐limited.^[^ ^9^ ^]^ In addition, nanosheet networks tend to be electrically limited by their junction resistance due to poor alignment of nanosheets.^[^ ^19a^ ^]^ These junctions may act as recombination centers that reduce the photocarrier lifetime and limit the carrier diffusion length.^[^ ^12^ ^]^ Therefore, a thinner nanosheet network is desirable rather than a thicker one.

This study aims to realize TMDs‐based, vertically stacked, heterojunction diodes where all non‐electrode layers are solution‐processed. WSe_2_ was liquid‐exfoliated and used as a light‐absorbing layer, in combination with vertically stacked layers consisting of solution‐processed poly(3,4‐ethylenedioxythiophene) polystyrene sulfonate (PEDOT: PSS) and ZnO nanoparticle layers, and alongside non‐solution processed indium tin oxide (ITO) and aluminum (Al) electrodes. These vertical heterojunctions were fabricated using a combination of spray coating and spin coating techniques. The resultant heterojunctions showed a high rectification ratio of ≈10^4^ at ±1 V. Under illumination, the device could be operated as a photodiode at zero (self‐powered) and reverse biases, and a photoconductor at forward biases.

## 2 Results and Discussion

### 2.1 Material and Device Characterizations

**Figure 1:**
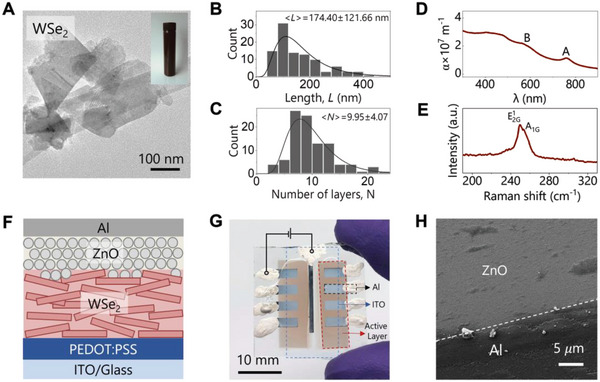
A) A TEM image of liquid exfoliated WSe_2_ nanosheets, and the inset is a photograph of a stable WSe_2_ IPA dispersion. B,C) are AFM statistical results of WSe_2_ nanosheets’ length *L* and number of layer *N* distributions, respectively. D) UV–vis absorption coefficient α spectrum of a sprayed WSe_2_ nanosheet film. E) Raman spectrum of a WSe_2_ film deposited on the SiO_2_/Si substrate. F) The schematic of the proposed device structure. G) A photograph of the fabricated devices, where the blue, red, and black dashed squares indicate ITO, active layers, and Al electrodes, respectively. H) A top‐view SEM image of the device.

WSe_2_ nanosheets were prepared by liquid phase exfoliation of the powder (Alfa Aesar, 99.8%) in deionized water and sodium cholate hydrate (SC, Sigma‐Aldrich, ≥99%) solution in an ultrasonic bath. The dispersion was purged with the high‐purity nitrogen during exfoliation to remove excess oxygen and prevent nanosheet oxidation. The size selection of nanosheets was performed by liquid cascade centrifugation^[^ ^21^ ^]^ to remove un‐exfoliated material as well as ultra‐small species. As water is problematic for film formation due to its high surface tension and boiling point,^[^ ^22^ ^]^ the SC/H_2_O dispersion was solvent‐exchanged to isopropanol (IPA, Sigma Aldrich, HPLC grade) by repeat centrifugation and re‐dispersing. The experimental details are presented in Figure 1 (Supporting Information). The final obtained WSe_2_/IPA dispersion with a concentration of ≈3 mg mL^−1^ (**Figure** **1**A inset).

The exfoliated nanosheets were characterized by transmission electron microscopy (TEM), and a typical TEM image of nanosheets is shown in Figure 1A. Plate‐like morphology can be observed, and their sizes range from ≈100 to 200 nm. To find the length and thickness distribution of these nanosheets, we performed atomic force microscopy (AFM) with nanosheets deposited on Si/SiO_2_ substrates by drop‐casting. More than 100 nanosheets were counted and the statistical results are given in Figure 1B,C, respectively. The mean length *<L>* and the number of layers *<N>* for nanosheets are 174 nm and 10, respectively, corresponding to a length‐to‐thickness aspect ratio of ≈27, which agrees with previously reported LPE TMDs nanosheets exfoliated in water/surfactant.^[^ ^21^, ^23^ ^]^

The WSe_2_ dispersion was spray‐coated onto a glass slide to yield a network of thickness ≈20 nm, which was then characterized by UV–vis spectroscopy. The absorption coefficient (*α*) spectrum is plotted in Figure 1D. The mean absorption coefficient of WSe_2_ in the visible regime (400 to 700 nm) is ≈2.14 × 10^7^ m^−1^ (characteristic absorption length, 1/*α* ≈ 50 nm) which is appropriate for optoelectronic applications. The characteristic excitonic absorption peaks A and B for WSe_2_ can be found at ≈760 and 590 nm, respectively. The dispersion was drop‐casted on a Si/SiO_2_ substrate and the film was characterized by Raman spectroscopy (Figure 1E). The Raman spectrum shows two distinct peaks corresponding to E^1^_2G_ and A_1G_ modes. The optical properties of the exfoliated WSe_2_ are consistent with previously reported results.^[^ ^24^ ^]^

Following the successful preparation of nanosheet dispersions, we now move on to heterojunction fabrication. We aim to realize a conventional diode structure, for example, cathode/hole transport layer (HTL)/light‐absorbing layer/electron transport layer (ETL)/anode. We use ITO‐coated glasses (Ossila, 20 Ω sq^−1^) as the bottom transparent electrodes, and choose PEDOT: PSS (Ossila, Al4083) as HTL, and ZnO nanoparticles (Sigma‐Aldrich, N‐11‐Jet, 8–16 nm) as ETL (TEM image is shown in Figure S1, Supporting Information). PEDOT: PSS and ZnO are established transport layer materials for various optoelectronic applications^[^ ^25^ ^]^ and can be simply solution‐processed into thin films. They have distinct energetic properties and low absorption coefficients in the visible regime compared with WSe_2_, allowing us to investigate the role of WSe_2_ nanosheet networks as the light‐absorbing layer in such hetero‐structured devices. In addition, assuming ZnO nanoparticles can realize full coverage on top of the porous nanosheet network they will help avoid metal diffusion into networks.

First, PEDOT: PSS was spin‐coated onto an ITO substrate and annealed to form a layer of ≈50 nm thick. Next, spray coating was adopted to uniformly deposit WSe_2_ nanosheet networks on the PEDOT: PSS layer, leading to a WSe_2_ layer ≈20 nm thick. The ZnO nanoparticle dispersion was spin‐coated onto the WSe_2_ film twice to achieve ≈55 nm thickness to maximize coverage. The device is completed by a thermally evaporated 100 nm aluminum (Al) top electrode through the shadow mask. The final device structure is ITO/PEDOT: PSS/WSe_2_/ZnO/Al as depicted in Figure 1F. More experimental details are presented in Figure S1 (Supporting Information). A representative photograph of the obtained device is shown in Figure 1G. The device area is defined as the overlapping area between ITO (blue dashed square) and Al (red dashed square), which is ≈6.5 mm^2^. Finally, silver paste was applied to each Al electrodes as well as ITO for electrical contacts.

The morphology of the fabricated devices was characterized by scanning electron microscopy (SEM). A representative top‐view SEM image is shown in Figure 1H. ZnO nanoparticles are uniformly coated on the WSe_2_ layer with the absence of pinholes, and a distinct edge between the Al electrode and ZnO film can be observed. The device ITO/PEDOT: PSS/WSe_2_/ZnO/Al was fractured after being frozen by the liquid nitrogen and its cross‐sectional SEM image is acquired, which is shown in Figure S2 (Supporting Information). There is no visible Al metal filament in contact with the PEDOT: PSS layer, confirming the successful fabrication of the proposed device structure.

### 2.2 Electrical Characterizations

**Figure 2:**
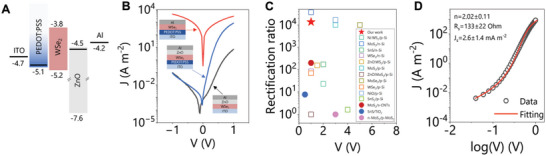
A) The energy band diagram of each material. B) The semi‐logarithmic *J–V* curves of three different devices by varying the transport layers. C) Comparison of rectification ratios at various biases from literature. n‐ and p‐ refer to n‐type and p‐type doping, respectively. Open symbols represent heterojunctions made from semiconducting nanosheet networks and heavily doped silicon. Closed symbols represent the diodes with all‐solution‐processed active layers. D) A representative fitting curve and the statistical fitting results are given in the figure.

The energy bands of each material used for the device are depicted in **Figure** **2**A. The work function (WF) of ITO is −4.7 eV.^[^ ^26^ ^]^ The conductive polymer PEDOT: PSS facilitates hole transport due to its deep WF of ≈−5.1 eV^[^ ^27^ ^]^ but can only weakly block electron transport.^[^ ^28^ ^]^ The reported conduction band minimum (CBM) of WSe_2_ ranges from −3.5 to −4 eV for few‐ and multi‐layered nanosheets.^[^ ^29^ ^]^ We estimate −3.8 eV as its CBM so, taking 1.4 eV as the bandgap,^[^ ^30^ ^]^ the valence band maximum (VBM) will be −5.2 eV. The Fermi level *E_f_* for WSe_2_ is unknown and will likely affected by processing details. However, it was previously reported to exhibit p‐type behavior in ambient conditions,^[^ ^20a^ ^]^ while n‐type conduction could dominate under lowered pressure conditions. It is found in the following electrical characterization section that there could be a potential barrier at the ITO/WSe_2_ interface for hole transport, indicating *E_f_* for WSe_2_ is probably deeper than −4.7 eV. The energy bands of ZnO were estimated by electrical characterizations on ITO/PEDOT: PSS/ZnO/Al Schottky diodes (Figure S3, Supporting Information). The extracted CBM is ≈−4.5 eV, similar to previous reports.^[^ ^32^ ^]^ As ZnO is a naturally n‐doped material due to oxygen vacancies, its *E_f_* will be very close to its CBM and is estimated to be −4.6 eV.^[^ ^33^ ^]^ Using optical bandgap *E*_g_ ≈ 3.1 eV determined from its absorption spectrum (shown in Figure S4, Supporting Information), the VBM will be ≈−7.6 eV. The top electrode is Al which has a WF of −4.2 eV and so can make Ohmic contact with ZnO.

To understand the role of each layer in our primary 3‐layer device (ITO/PEDOT: PSS/WSe_2_/ZnO/Al), we also characterize heterojunctions with structure: ITO/WSe_2_/ZnO/Al, and ITO/PEDOT: PSS/WSe_2_/Al. The thicknesses for each layer type in these devices were kept constant from device to device using identical coating parameters. All devices were deliberately left in the ambient overnight and then encapsulated simply by drop‐casting clear varnish consisting of nylon‐based polymer on the top to obtain consistent results (Figure S5, Supporting Information). The electrical properties of devices were investigated by collecting current‐voltage (*I–V)* characteristics from −1 to 1 V in ambient but dark conditions. The current was then converted into current density *J* by dividing the device area *A*. Their representative *J–V* curves are given in Figure 2B. The ITO/PEDOT: PSS/WSe_2_/Al device shows an almost symmetric *J–V* curve with *J* much larger than the other devices. However, these results may be misleading since Al metal atoms probably diffuse into pores or pinholes of WSe_2_ nanosheet networks and are likely in contact with metallic PEDOT: PSS during the thermally evaporating top electrode process.^[^ ^9^, ^19^ ^]^ This highlights the difficulty in working with porous nanosheet networks. Such electrical shorts can be avoided by coating ZnO nanoparticles on top of WSe_2_. For example, a clear rectifying behavior was observed for ITO/WSe_2_/ZnO/Al with a rectification ratio (RR) at ±1 V of ≈73 (Full *J–V* curves and analysis are in Figure S6, Supporting Information). When both PEDOT: PSS and ZnO are used in the full device stack (ITO/PEDOT: PSS/WSe_2_/ZnO/Al), *J* at forward bias is significantly larger than that from ITO/WSe_2_/ZnO/Al devices. A stronger rectification was observed with an RR of ≈10^4^ (Full *J–V* curves are in Figure S7, Supporting Information).

To understand their electrical behaviors, the WSe_2_/ZnO interface is assumed to act as a type‐2 heterojunction. Without external biases, once WSe_2_ and ZnO are in contact and form an interface with an internal built‐in potential *Φ_b_*, the Fermi level will be constant throughout WSe_2_ and ZnO under thermal equilibrium. Therefore, electrons diffuse from ZnO into WSe_2_ and holes diffuse in the opposite direction, forming depletion regions extending from the interface into both semiconductors up to certain thicknesses defined by their doping densities. As a result, we expect a CBM offset ΔCBM of ≈0.7 eV and a VBM offset ΔVBM of ≈2.4 eV formed at the WSe_2_/ZnO interface.

Here, only devices with ZnO ETLs are discussed. Under small forward biases, electrons have insufficient energy to overcome ΔCBM from ZnO to WSe_2_. With increasing forward bias, the reduction of ΔCBM greatly improves electron injection, resulting in an exponential increase of *J*. Under higher forward biases, ΔCBM is compensated by the bias, and *J* becomes linear as the device becomes limited by its series resistance. From the *J–V* curves in Figure 2B, the forward‐bias *J* from the device without PEDOT: PSS is much lower than the one with PEDOT: PSS. This is due to a Schottky barrier formed at the ITO/WSe_2_ interface that blocks hole transport from WSe_2_ to ITO. Introducing PEDOT: PSS at this interface can reduce or even remove the potential barrier and allows facile hole injection, eventually yielding a higher *J* at forward biases. Under reverse biases, both heterojunction devices gave similar small *J*. The small reverse current density could be attributed to the prohibited hole transport from WSe_2_ to ZnO due to the large ΔVBM formed at this interface. Therefore, this analysis indicates that rectifying behavior would mainly come from the WSe_2_/ZnO interface.

The presence of the p‐n junction and the electron‐ and hole‐injecting electrodes means the ITO/PEDOT: PSS/WSe_2_/ZnO/Al heterojunction device works effectively as a diode with much higher forward than reverse bias as mentioned above. We quantify this via the RR at ±1 V, of which our best device displayed RR = 1.4 × 10^4^ which is among the highest reported for such devices. We summarize the reported solution‐processed diodes incorporating LPE TMDs nanosheets in Table S1 (Supporting Information) and plot their RR against various biases in Figure. 2C. The open symbols represent RR from diodes using nanosheets deposited on heavily doped silicon, and closed symbols represent diodes with all‐solution‐processed active layers. The RR from our device is shown in red star and is higher than all but one TMDs/Si device.

The current density of a heterojunction formed at the WSe_2_/ZnO interface can be described by the Shockley equation,^[^ ^34^ ^]^ which can be modified to account for the voltage drop across a series resistance, *R_s_*, representing the resistance of the active layers:

(1) $$J=J_{s}\exp\left[\left.\frac{q(V-JR_{s}A)}{nkT}\right]\right.$$

where *J_s_* is the saturation current density, *n* is the ideality factor, *q* is the elementary charge, *k* is the Boltzmann constant, and *T* is the temperature.

To extract the electrical parameters of the fabricated devices, the *J–V* data was fitted using Equation (1).^[^ ^9^ ^]^ This procedure gave good fits, yielding the diode parameters *n*, *J_s_*, and *R_s_*, with one example shown in Figure 2D (all fittings are shown in Figure S7, Supporting Information). Generally, the ideality factor is usually in the range of 1–2, where *n* = 1 accounts for diffusion current‐dominated conduction and *n* = 2 stands for generation/recombination‐dominated conduction mechanism.^[^ ^34^ ^]^ The obtained *n* is ≈2, indicating these devices are dominated by generation/recombination.

The obtained *R_s_* is 133 ± 22 Ohm, which includes the resistance of the WSe_2_ network along the out‐of‐plane (OoP) direction as well as that of the other materials. The OoP resistance of WSe_2_ is obtained by subtracting the series resistance of the ITO/PEDOT: PSS/ZnO/Al device from that of the full 3‐layer device. The resultant resistance yields an OoP conductivity of WSe_2_ *: σ_OoP,WSe2_* = 7.55 × 10^−5^ S m^−1^. This value is roughly one order of magnitude lower than its in‐plane (IP) value which tends to be in the range 10^−4^ to 10^−3^ S m^−1^.^[^ ^20^, ^35^ ^]^ This low electrical anisotropy (IP to OoP) is consistent with our previous study on LPE semiconducting nanosheet networks in a random stacking arrangement.^[^ ^9^ ^]^

Lower *J_s_* values imply a lower recombination current, leading to a better performance for applications such as solar cells. The extracted *J_s_* is 2.6 ± 1.4 mA m^−2^. Our value is orders of magnitude larger than solution‐processed solar cells, fabricated from organics,^[^ ^36^ ^]^ quantum dots,^[^ ^37^ ^]^ and perovskites.^[^ ^38^ ^]^ Therefore, our device suffers from significant recombination associated with either the heterojunction interface or the nanosheet network.

### 2.3 Electrical Characterizations under Illumination

**Figure 3:**
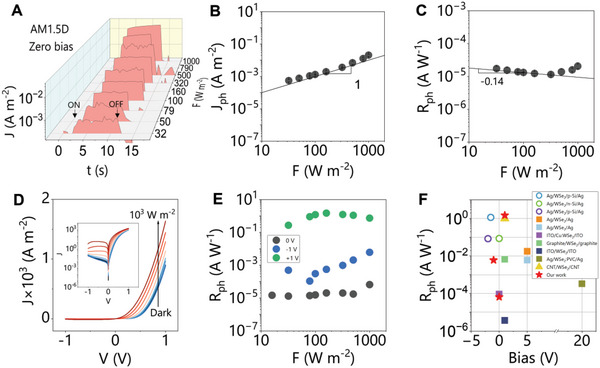
A) The temporal *J_ph_* responses of the device ITO/PEDOT: PSS/WSe_2_/ZnO/Al upon AM1.5D illumination under various light intensities without external biases. B,C) are *F*‐dependent *J_ph_* and *R_ph_* extracted from (A), respectively. D) The collected *J–V* curves from dark to 10^3^ W m^−2^. The inset is its semi‐logarithmic plot. E) *F* dependent *R_ph_* extracted from (D) at different biases (−1, 0, and 1 V). F) Comparison of *R_ph_* at different biases from LPE WSe_2_ nanosheets based devices. The round open circles represent heterojunction‐type devices, and solid symbols represent Ohmic‐contacted devices with squares representing the lateral device structure and triangle representing the vertical device structure.

Given the observed diode behavior of these devices, optoelectronic responses of all three types of devices are measured under a solar simulator (Newport 96 000) equipped with an AM1.5D filter. The light intensity *F* was tuned using a shutter and neutral density filters, varying from dark to 1000 W m^−2^. The temporal behavior of photocurrent *I_ph_* under illumination was recorded without any external bias (photocurrent is illuminated current minus dark current). A shutter was manually placed and removed on top of the device to simulate the light “on” and “off” states. The obtained photocurrent was then divided by device area *A* to obtain photocurrent density *J_ph_*. The temporal *J_ph_* responses from ITO/PEDOT: PSS/WSe_2_/ZnO/Al at various light intensities are shown in **Figure** **3**A. The device exhibited obvious photoresponses with rise and decay times both less than a second (limited by the equipment resolution ≈450 ms). Because no bias was applied, the obtained *J_ph_* can be regarded as the short‐circuit current density *J_sc_*. This means that the device can be operated as a self‐powered photodetector. For completeness, the photoresponses for the other two types of devices were also measured. Under the same measurement conditions, there was no measurable photocurrent. This phenomenon verifies that the WSe_2_/ZnO interface indeed forms a built‐in potential and a depletion region that could separate photo‐carriers. Moreover, the PEDOT: PSS layer can remarkably reduce the recombination at the ITO/WSe_2_ interface and therefore enhance the photocurrent.

The *F*‐dependent *J_ph_* is extracted from Figure 3A and is shown in Figure 3B. *J_ph_* increases with *F* with an exponent very close to unity, consistent with the presence of only very shallow traps.^[^ ^12a^ ^]^ One important parameter to evaluate the photodetector performance is the photo‐responsivity, *R_ph_*, which is defined as*R_ph_* = *J_ph_*/*F*. Thus, using averaged data in Figure 3B, the *F*‐dependent *R_ph_* can be obtained and is given in Figure 3C. *R_ph_* decreases very weakly with *F* with an exponent of ≈−0.14 at relatively low *F*. A slight increase of *R_ph_* is observed at higher *F* probably due to thermally generated current. These values of *R_ph_* will be compared to the literature below.

The *J–V* curves under various *F* were also collected and are shown in Figure 3D. The semi‐logarithmic plot is given in its inset. The linear *J–V* curves show obvious photoresponses at forward biases, while the semi‐logarithmic curve shows that photoresponses can also be observed at reverse biases. This indicates that the fabricated photodetector can not only work in photodiode mode (at zero and reverse biases) but also in photoconductor mode (at forward biases). The short‐circuit current and open circuit voltage were not so obvious but were small but detectable (Figure S8, Supporting Information).

*J_ph_* values were extracted from the *J–V* curves at −1, 0, and 1 V and converted into *R_ph_*, which is plotted against *F* in Figure 3E. This plot directly compares the photodetector performance operated in photodiode and photoconductor modes. *R_ph_* at 0 V is consistent with the result from temporal *J_ph_* measurement (Figure 3C), which is low, ≈10^−5^–10^−4^ A W^−1^. It is worth noting that when there is no external bias, only photons absorbed in or within a diffusion length from the depletion region in either WSe_2_ or ZnO will be converted into photocarriers that can contribute to *J_ph_*. When the device is reverse biased (−1 V), the depletion width will be extended, and it allows more photons being absorbed in the depletion region. This is consistent with our observation that *R_ph_* at −1 V is at least 1 order of magnitude higher than that at 0 V. Interestingly, *R_ph_* at 1 V is more than 4 orders of magnitude higher than it at 0 V, indicating that the photoconductor mode is superior to the photodiode mode. Moreover, *R_ph_* at −1 and 0 V increase at *F* above ≈100 W m^−2^, indicating the presence of thermal effects.

The fact that *R_ph_* is higher at −1 than 0 V, implies that the depletion width is smaller than its film thickness such that the reverse bias can further widen the depletion width. This means that even though the WSe_2_ layer is thin (≈20 nm), it likely contains two regions, a normal region and a depleted region in contact with ZnO. This depleted region enables the device to work as a photodiode at zero and further at reverse biases. The photo‐carriers generated in this region will be driven by the built‐in potential and diffuse toward opposite electrodes. In the normal region, illumination will decrease its resistance via standard photoconductivity processes^[^ ^12b^ ^]^ so that the device can be operated as a photoconductor at positive biases. However, the obtained photocurrent density at 0 V is still significantly lower than the approximated theoretical value (Supporting information S11), implying that further improvements are expected in the future.

The observed phenomena may be associated with various factors. First, as the device was illuminated on the ITO side, the un‐depleted, normal region experienced light first. This may also favor the photo‐conductor mode rather than the photo‐diode mode. Second, as these nanosheets are mostly few‐layered, energy band variation across the network could trap the photo‐carriers and limit the diffusion length. Last, although no dopant was added to the dispersion on purpose and the surfactant was removed before film formation, some dopants from either trace amounts of residual surfactants^[^ ^9^ ^]^ or O_2_ and H_2_O in the air^[^ ^39^ ^]^ are inevitable. This un‐intentional doping effect could make the depletion width on the WSe_2_ side narrower than expected.

To compare our photodetector performance, the literature *R_ph_* values from LPE WSe_2_‐based devices were plotted against various biases to distinguish different photodetector operation modes in Figure 3F. These values are presented in Table S2 (Supporting Information). The open circle symbols represent the devices using heavily doped silicon substrates as one of the active layers, and these devices mainly worked in photo‐diode mode and showed better performance than ours at reverse biases. This result is not a surprise since silicon‐only diodes can be used as good photodetectors due to their thickness and high quantum efficiency,^[^ ^40^ ^]^ while photoresponses from our devices mainly come from the WSe_2_ layer alone. The solid squares represent Ohmic contacted devices fabricated in a lateral structure, and these devices can only be operated in photoconductor mode due to the lack of built‐in potential. Our device working as a photoconductor (positive bias) gives the highest *R_ph_* to be ≈1.5 A W^−1^ at 1 V. The only *R_ph_* which is close to our result at 1 V is shown in the solid triangle. It refers to a vertically structured and Ohmic contacted device that WSe_2_ layer was sandwiched between two metal electrodes made from carbon nanotubes. The large *R_ph_* probably comes from a much‐reduced channel length in a vertical structure compared with that in a lateral one.

To explore the operating mechanism of the obtained device, *F*‐dependent *J–V* curves were fitted by modifying Equation (1) to include a photoinduced current density *J_ph_*.^[^ ^41^ ^]^ When the device is not biased, *J_ph_* is equivalent to *J_sc_*.

(2) $$J=J_{ph}-J_{s}\left[\exp(\frac{q(V-JR_{s}A)}{nkT})-1\right]$$

**Figure 4:**
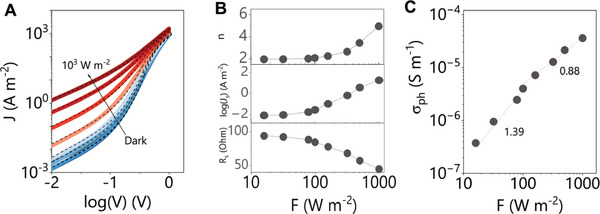
A) The *F*‐dependent *J‐log(V)* curves of ITO/PEDOT: PSS/WSe_2_/ZnO/Al and their corresponding fittings (dash line). B) The obtained fitting results, such as *n*, *J_s_*, and *R_s_* are presented from top to bottom against *F*, respectively. C) *σ_ph_* is extracted from *R_s_* shown in (B) bottom and is plotted against *F*.

This equation fits the data very well as shown in **Figure** **4**A, with the extracted parameters plotted versus *F* in Figure 4B and in the supporting information. Both *n* and *J_s_* remain almost constant at *F* ≤ ≈100 W m^−2^, but increase significantly when *F* exceeds this critical value. *n* remains ≈2 at *F* ≤ ≈100 W m^−2^, indicating the device is dominated by the recombination in the depletion region under relatively lower *F*. Above ≈100 W m^−2^, thermal effects increase *J_s_* by ≈4 orders of magnitude, and *n* increases to ≈5 at *F* = 1000 W m^−2^ possibly due to the involvement of the tunneling processes.^[^ ^42^ ^]^ Incidentally, this leads to even poorer photovoltaic performances as shown in Figure S8B (Supporting Information). The series resistance, *R_s_*, decreases with *F*, consistent with a positive photoconductivity *σ_ph_* of the device. Assuming the photoconductivity is associated with the WSe_2_ layer, *σ_ph_* is calculated using $\sigma_{ph}\left(F\right)=\left(l/A\right)\left[R_{s}^{-1}\left(F\right)-R_{s}^{-1}\left(F=0\right)\right]$ where *l* and *A* are the thickness and area of the WSe_2_ layer. The resultant photoconductivity is plotted versus *F* in Figure 4C. The lowest observed photoconductivity value was ≈4 × 10^−7^ S m^−1^ at 15 W m^−2^. In comparison, the *σ_ph_* of ITO/ZnO/Al was measured to be 3.46 × 10^−8^ S m^−1^ at 1000 W m^−2^ and a bias of 1 V, which is significantly lower than that for the whole device. Therefore, *σ_ph_* mainly comes from the WSe_2_ layer. In Figure 4C, *σ_ph_* increases as a power law with *F* (σ_*ph*_∝*F* ^β^), showing two regimes separated at *F* ≈ 100 W m^−2^. At relatively low *F*, *σ_ph_* exhibits superlinear dependence with *β* to be 1.39. *β* varies to 0.88 while *F* is above 100 W m^−2^, corresponding to a slightly sublinear dependence.

Sublinear dependence of *σ_ph_* on *F* was often observed in solution‐processed semiconducting nanosheet networks owing to the presence of trap states which increase the recombination.^[^ ^12^, ^43^ ^]^ Since thermal effects are usually more obvious at higher *F*, this phenomenon is consistent with previous reports. On the other hand, superlinear dependence is infrequently observed^[^ ^44^ ^]^ and it arises from an increase in the majority carrier lifetime with *F*. In other words, the device becomes more photo‐sensitive with increasing *F*, which can be contrasted with decreased carrier lifetime from sublinear dependence (less sensitive with *F*). Superliner behavior has been ascribed to two types of recombination centers with different capture cross‐sections for electrons and holes. One could act as sensitizer centers that prolong the carrier lifetime while the other one could induce recombination.^[^ ^45^ ^]^ Increasing *F* results in a shift of recombination center from one to the other, therefore showing different dependence behaviors. However, our measurement is inadequate to distinguish the pure photo effect from a photothermal one. The complexity of the nanosheet composition and network morphology could also contribute to the phenomena, and more measurements and characterizations are required.

## 3 Conclusion

In this study, we have successfully fabricated solution‐processed heterojunctions based on LPE WSe_2_ nanosheet networks where their active layers are all solution‐processed. The obtained devices show a high rectification ratio of up to ≈10^4^ at ±1 V. It can be used as a self‐powered photodetector that can be operated in both photodiode mode and photoconductor mode. *R_ph_* in photoconductor mode is the highest for LPE WSe_2_‐based photodetectors and is ≈1.5 A W^−1^ at 1 V. However, *R_ph_* obtained in photodiode mode is still lower than that from silicon‐based heterojunctions.

## Conflict of Interest

The authors declare no conflict of interest.

## Supporting information

Supporting Information

## Data Availability

The data that support the findings of this study are available from the corresponding author upon reasonable request.
