# Supplementary material for: Solution‐Processed Heterojunction Photodiodes Based on WSe2 Nanosheet Networks
Source: Small. 2023 Sep 21;21(28):2304735. doi: 10.1002/smll.202304735 (PMC12272024; doi:10.1002/smll.202304735)
Supplement: Supplementary file 1 — Supporting Information [file SMLL-21-2304735-s001.pdf]

NANO · MICRO small

Supporting Information

for *Small*, DOI 10.1002/smll.202304735

Solution-Processed Heterojunction Photodiodes Based on WSe_2 Nanosheet Networks

*Shixin Liu, Tian Carey, Jose Munuera, Kevin Synnatschke, Harneet Kaur, Emmet Coleman, Luke Doolan and Jonathan N. Coleman**

Solution-processed heterojunction photodiodes based on WSe_2 nanosheet networks

Shixin Liu, Tian Carey, Jose Munuera, Kevin Synnatschke, Harneet Kaur, Emmet Coleman, Luke Doolan, Jonathan N. Coleman*

S. Liu, T. Carey, J. Munuera, K. Synnatschke, H. Kaur, E. Coleman, L. Doolan, J. N. Coleman
School of Physics, CRANN & AMBER Research Centres, Trinity College Dublin, Dublin 2, Ireland.

J. Munuera
Department of Physics, Faculty of Sciences, University of Oviedo, C/ Leopoldo Calvo Sotelo, 18, 33007 Oviedo, Asturias, Spain

*E-mail: colemaj@tcd.ie; Tel: +353 (0) 1 8963859

S1 Experimental

Material preparation:

The WSe_2 nanosheet dispersion is obtained by liquid phase exfoliation in an inert atmosphere. WSe_2 powder (Alfa Aesar, metal basis) is mixed in 2 mg/mL sodium cholate hydrate (Sigma-Aldrich, ≥99%) aqueous (SC/H_2O) solution at a concentration of 30 mg/mL. The powder dispersion was contained in a glass flask with a rubber cap. Nitrogen was injected into the dispersion continuously through a needle to maintain the inert atmosphere. As a pre-treatment step to remove any impurities in the powder, the dispersion was bath-sonicated for 1 h and was centrifuged at 6 krpm for 1 h. The sediment was kept and re-dispersed in a fresh 6 mg/mL SC/H_2O solution. This dispersion was again under bath sonication for another 8 h with a continuous nitrogen flow. The water in the sonic bath was replaced with ice-cooled water every hour to avoid over-heating.

The obtained dispersion from the last step contains poly-dispersed nanosheet with a broad distribution of size and thickness. Thus, a size-selection step is introduced by liquid cascade centrifugation (LCC). The dispersion was centrifuged at 2 krpm for 2 h and the supernatant was collected so that the large and thick partially-exfoliated nanosheet was removed. The supernatant was centrifuged again at 6 krpm and the sediment was collected to remove the ultra-small and thin nanosheets.

As water is a troublesome for device fabrication due to its high boiling point, the obtained sediment was dispersed and washed in isopropanol (IPA, Sigma Aldrich, HPLC grade) by centrifuging and decanting twice. The WSe_2 IPA dispersion was finally obtained and used for further characterization and device fabrication.

Device fabrication:

Substrate treatment

Indium tin oxide (ITO) coated glass slides (Ossila, 100 nm thick, 20 Ω sq^{-1}) were used as bottom electrodes. The ITO glass was cut into 2.5 cm×2.5 cm and Kapton tape was

used to cover each substrate with an area of 2.5 cm×1.3 cm. The covered ITO glass was immersed into 2 M hydrochloric solution at 80 °C for 5 min to etch the exposed ITO area. The obtained ITO glass was cleaned in ~5 vol% Hellmanex/H_2O solution, H_2O, and IPA in sequence for 10 min each. Then, ITO glass was blow-dried by a compressed nitrogen flow and was under oxygen plasma treatment for 5 min at a power of 100 W.

Heterojunction devices

For heterojunction devices, ITO glasses were used as bottom electrodes as well as the substrates. All the solution processes were done in ambient. Poly(3,4-ethylenedioxythiophene) polystyrene sulfonate (PEDOT:PSS) aqueous solution (Ossila, Al4083) was filtered through 0.45 μm PTFE filters to remove aggregates. The filtered PEDOT:PSS was spin-coated onto ITO at a speed of 3000 rpm for 40 s using a spin coater (Model: WS-400A-8NPP/LITE). The ITO/PEDOT:PSS was annealed at 140 °C for 20 min. The mean thickness of PEDOT:PSS layer was ~50 nm. WSe_2 IPA dispersion was then sprayed on ITO/PEDOT:PSS and annealed in glovebox at 100 °C for 30 min. The mean thickness of the WSe_2 layer is 20 ± 8 nm. ZnO nanoparticles in IPA and propylene glycol (Sigma-Aldrich, N-11-Jet, particle size: 8-16 nm, 2.5 wt%) was diluted in IPA at a volume ratio of 1:1 and was bath-sonicated for 10 min before use. It was spun onto ITO/PEDOT:PSS/WSe_2 at a speed of 2000 rpm twice and annealed in ambient at 80 °C for 10 min. The mean thickness of ZnO layer was ~55 nm. The top electrode aluminum (100 nm) was evaporated by Temescal FC-2000 through a shadow mask at a rate of 0.2 nm per second and the final device structure was ITO/PEDOT:PSS/WSe_2/ZnO/Al. The completed devices were annealed in glovebox for 1 h at 80 °C to improve the contact. For ITO/PEDOT:PSS/WSe_2/Al and ITO/WSe_2/ZnO/Al, they were fabricated in the same way. The device area was defined as the overlapping area between top and bottom electrodes.

Encapsulation

We noticed that the electrical behaviors of heterojunctions could change significantly in ambient within a few hours. Given the measurement were performed in ambient, we deliberately left devices in air overnight to give stabilized performance. The "aged" devices were encapsulated by drop-casting a transparent varnish (Sally Hansen). The devices were again left in air overnight to allow the varnish to dry completely before measurements.

Characterizations

UV-Vis absorption spectroscopy

The transmittance spectra of the WSe_2 film on a glass slide and a glass slide alone were recorded by Cary 50 spectrophotometer in the range of 300-900 nm. The transmittance T was converted into absorbance A using formula $A = -\lg(T)$. The absorbance of WSe_2 was obtained by $A_{WSe_2} = A_{Total} - A_{Glass}$. The absorption coefficient α was calculated by $\alpha = 2.303A/t$, where t is the film thickness.

Raman spectroscopy

WSe_2 ink were drop-casted onto Si/SiO_2 substrate and the obtained film was annealed at 120°C. The Raman spectrum of the film was acquired with a WITec Raman system with a 20× objective at a laser wavelength of 633 nm. The final spectrum is an average of 10 accumulations.

Atomic force microscopy

A Bruker Multimode 8 atomic force microscope was employed in ScanAsyst mode using an OLTESPA R3 cantilever. The nanosheet dispersion were drop-casted on SiO_2/Si substrates. More than 100 nanosheets were scanned and statistically analyzed.

Transmission electron microscopy

A JEOL 2100 microscope was used for imaging the nanoparticles. It was operated at an accelerating voltage of 200 kV. For sample preparation, ~10 μL dispersion was

drop-casted onto the Holey carbon grids (400 mesh). The grids were then dried at 70 °C under vacuum overnight before TEM observation.

Scanning electron microscopy

The SEM image was obtained by a Zeiss Ultra Plus at an accelerating voltage of 2 kV.

Profilometry

The thicknesses of films were measured by a Bruker Dektak profilometer. The line profile was taken with 1000 μm length and a resolution of ~50 nm/sample. More than 4 profiles were taking for each sample. The stylus force was set to 1 mg. The thickness of each layer was determined on another identical film fabricated in the same way on pre-cleaned glass substrates.

Electrical measurement

The current-voltage (*I-V*) characteristics were collected by Keithley 2400 from -1 V to 1 V with a step interval of 50 ms in the dark. The measurement under illumination was conducted with a solar simulator (Newport 96000) equipped with an AM1.5D filter. The light intensity of the solar simulator was calibrated by a reference silicon solar cell (RQS3677) with an effective illumination area of 2 cm × 2 cm. All electrical measurements under illumination were conducted without additional masks to define the illumination area. As the active area of the reference photodiode (4 cm^2) is much larger than the device area, we used the device area as the illumination area. To vary the light intensity, several neutral density filters were used. To record the temporal photo-responses, a metal foil was used as a shutter and was manually placed and removed on top of the device to simulate light "on" and "off" states.

S2 TEM image of ZnO nanoparticles

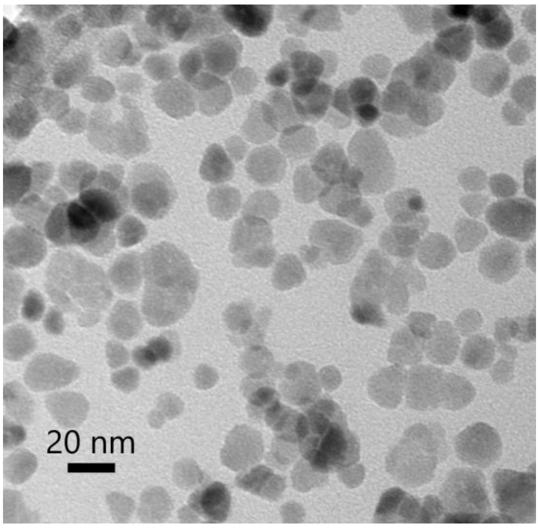

Fig. S1 TEM image of ZnO nanoparticles.

S3 Cross-section of the heterojunction.

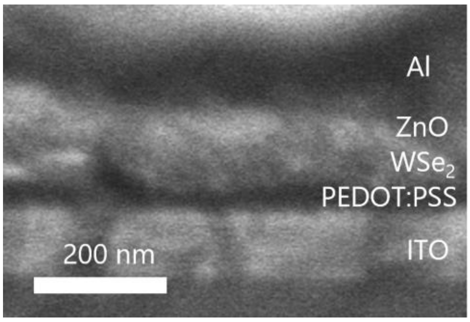

Fig.S2 A cross-sectional SEM image of ITO/PEDOT:PSS/WSe_2/ZnO/Al.

The entire substrate was immersed into acetone for 10 min at room temperature to dissolve the encapsulation layer. The substrate was fractured after frozen it in the liquid nitrogen. The cross-section was checked under SEM (Zeiss Ultra Plus), which is shown in Fig. S2.

S4 Electrical characterizations on the ITO/PEDOT:PSS/ZnO/Al Schottky diodes

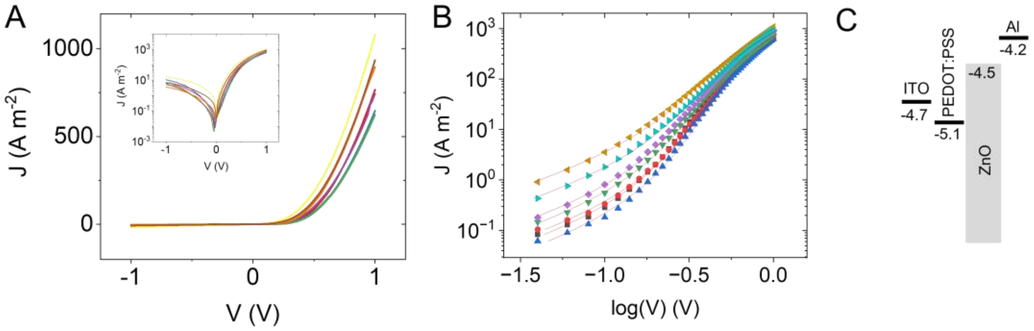

Fig. S3 (A) *J-V* curves of ITO/PEDOT:PSS/ZnO/Al with the semi-log plots in its inset. (B) Fittings of the *J-V* curves and (C) Energy band of each material.

ITO/PEDOT:PSS/ZnO/Al Schottky type diodes were fabricated following the procedure described in S1. These devices show rectifying behaviors with a rectification ratio at ±1 V around 100 (Fig. S3A). The electrical properties were extracted using Shockley equation (Fig. S3B). However, the saturation current density J_s for Schottky devices is described by:

$$J_s = A^* T^2 \exp(-\frac{q\phi_b}{kT}) \quad (1)$$

where ϕ_b is the potential barrier at the PEDOT:PSS/ZnO interface, which corresponds to the difference between the CBM of ZnO and WF of PEDOT:PSS. A^* is the Richardson constant. The fittings are shown in Fig. S3B. The obtained ideality factor n is 2.81±0.33, series resistance R_s is 92.71±14.85 Ohm, and ϕ_b is 0.65±0.03 eV taking the effective electron mass m^* in ZnO is $0.3m_0$.[1]

The energy band of each material in depicted in Fig. S3C. Given the WF of PEDOT:PSS is -5.1 eV, we can find the CBM for ZnO is ~-4.5 eV.

S5 Absorption coefficient spectrum of a ZnO thin film

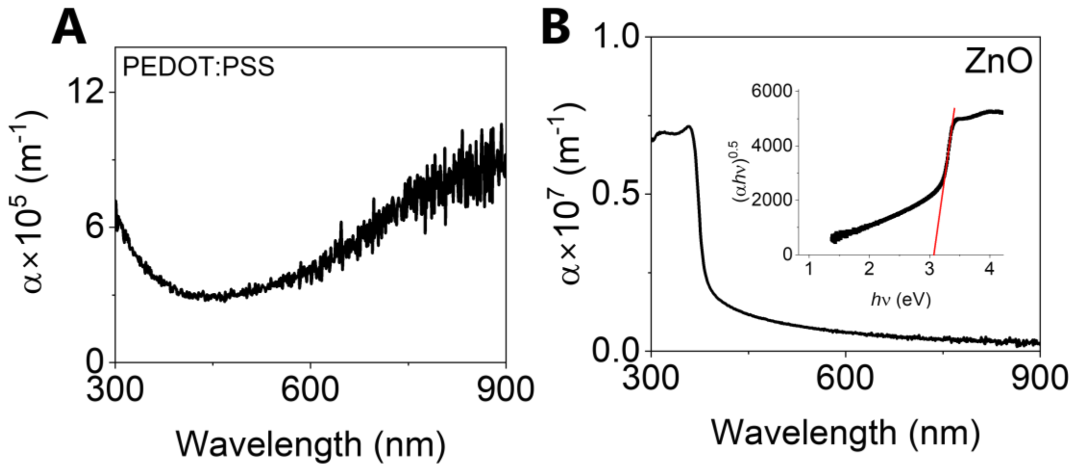

Fig. S4 Absorption coefficient spectra of spin-coated PEDOT:PSS (A) and ZnO (B) thin films on a glass substrate. The inset in B is the Tauc plot to extract its optical bandgap.

The PEDOT:PSS and ZnO thin films were separately spin-coated on a solvent-cleaned and oxygen-plasma treated glass slide. The film thicknesses for PEDOT:PSS and ZnO are around 50 and 55 nm, respectively. The absorption coefficient spectra are obtained following similar method for WSe_2 and shown in Fig. S4, which is detailed in the experimental section. The absorption coefficients of PEDOT:PSS and ZnO are much smaller than WSe_2 in visible regime as expected. The optical bandgap is obtained by a Tauc plot in Fig. S4B inset. A cutoff to the absorption edge gives a bandgap of around 3.1 eV.

S6 *J-V* curves of diodes left in ambient

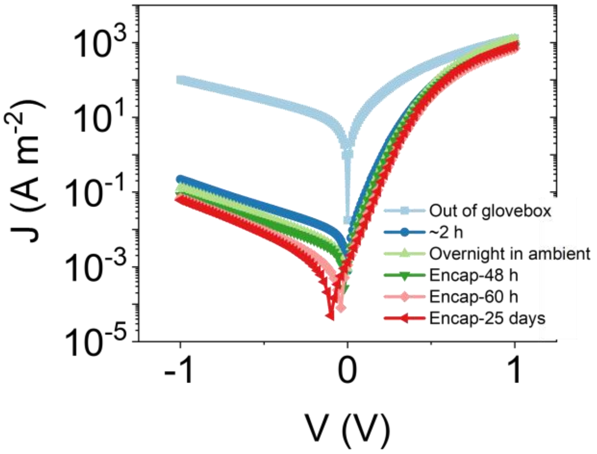

Fig S5. The *J-V* curves of ITO/PEDOT:PSS/WSe_2/ZnO/Al devices measured after taking out of glovebox, left in ambient for ~2 hours and overnight, and after encapsulation for a few hours to a few days.

The *J-V* curves were collected in ambient conditions. We found that the reverse current density would decrease significantly after being taken out from the glovebox and left in ambient within a few hours. This behavior may be associated with adsorption of water and oxygen from the air and results in doping of materials or a change in interface properties. However, since we performed most of the fabrication and measurement in the air, we decide to "age" all the devices on purpose to give consistent measurements. The "aged" devices were encapsulated by a commercially available transparent varnish from Sally Hansen and left in air to dry naturally. Such "aged and encapsulated" devices could maintain their electrical properties for more than a month in ambient. It is expected that more investigation is required on the aging phenomena of devices in the future.

S7 Electrical characterizations on ITO/WSe_2/ZnO/Al

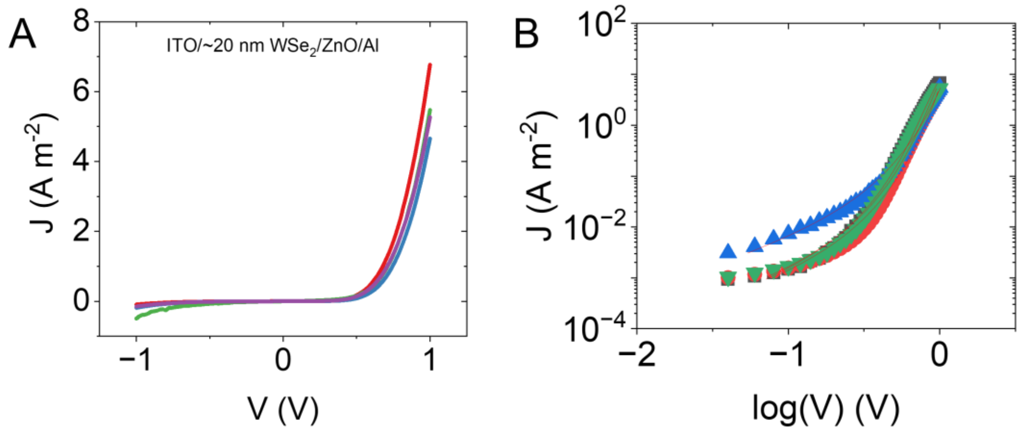

Fig. S6 *J-V* curves (A) and their fittings (B) of ITO/WSe_2/ZnO/Al, where the WSe_2 thickness is around 20 nm.

The *J-V* curves of ITO/WSe_2/ZnO/Al exhibit rectifying behaviors and we can use Shockley equation to fit the curves. The obtained ideality factor n, saturation current density J_s, and series resistance R_s are 4.78±0.92, $2.57\times10^{-3}\pm3.11\times10^{-3}$ A/m^2, and 1205±331 Ohm, respectively. The ideality factor is larger than the ITO/PEDOT:PSS/WSe_2/ZnO/Al with a comparable WSe_2 thickness. J_s is slightly lower and R_s is significantly larger. This phenomenon is likely associated with imperfect Ohmic contact at the ITO/WSe_2 interface. A potential barrier is formed and thus induces higher contact resistance as well as the poor diode behaviors.

S8 *J-V* curves of ITO/PEDOT:PSS/WSe_2/ZnO/Al

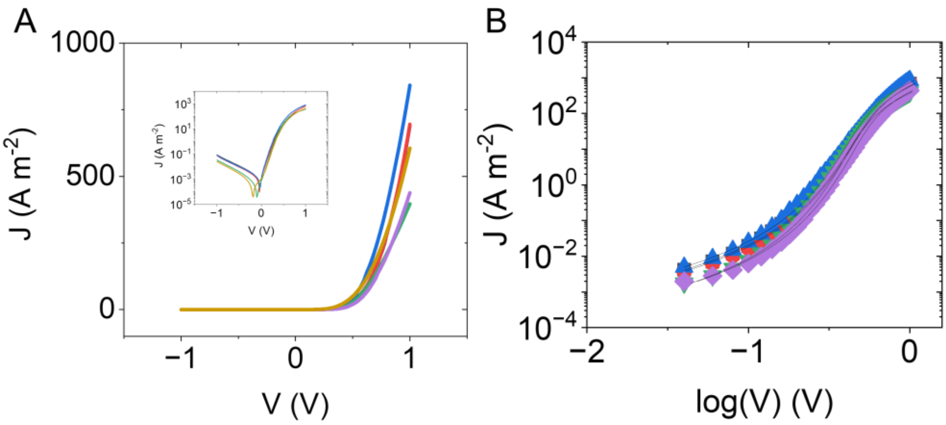

Fig. S7 *J-V* curves (A) and their fittings (B) of ITO/PEDOT:PSS/WSe_2/ZnO/Al.

S9 Comparison of rectification ratios at various biases

Table S1 Rectification ratios at various biases from the reported planar heterojunctions using liquid exfoliated TMD nanosheets.

No.	Material	Method	Structure	Rectification ratio	Ref.
1	MoS_2	Spray coat	In/n-Si/MoS_2/Au	*3.7e4 at ±1 V	[2]
2	WSe_2	Drop cast	Ag/WSe_2/n-Si/Ag	84.06 at ±1 V	[3]
3	WS_2	Drop cast	Al/ZnO:WS_2/p-Si	155 at ±1.5 V	[4]
4	MoS_2	Spin coat	Au/MoS_2/p-Si/Al	1e5 at ±3 V	[5]
5	MoS_2	Drop cast	Graphite/ZnO:MoS_2/p-Si/Graphite	*1 at ±1 V	[6]
6	$MoSe_2$	Spin coat	Au/$MoSe_2$/p-Si/Al	1e5 at ±5 V	[7]
7	WSe_2	Drop cast	Ag/WSe_2/p-Si/Ag	69 at ±1 V	[8]
8	WS_2	Drop cast	Ag/Ni:WS_2/p-Si/Ag	23 at ±2 V	[9]
9	SnS_2	Drop cast	Ag/SnS_2/p-Si/Ag	*1.62 at ±4 V	[10]
10	MoS_2	Spin coat	ITO/n-MoS_2/p-MoS_2/Ag	*1 at ±1 V	[11]
11	EE MoS_2	Spin coat	Graphene/s-CNTs/MoS_2/HfO_2/Graphene	200 at ±1 V	[12]
12	NiO	Spin coat	Ni/NiO/p-Si/InGa	16827 at ±3 V	[13]
13	SnS	Drop-cast	Ag/SnS/n-Si/Ag	171 at ±2 V	[14]
14	SnS	Dip coat	Pt/SnS/TiO_2/FTO	7.65 at ±0.5 V	[15]
15	WSe_2	Spray coat	ITO/PEDOT:PSS/WSe_2/ZnO/Al	13699 at ±1 V	Our work

Note: “Method” refers to the film formation method for LPE materials. “n-” and “p-” refer to n-type and p-type doping, respectively. “Gra” refers to graphite. “FTO” refers

to Fluoride-doped tin oxide glasses**.** "EE" refers to electrochemically exfoliated. The symbol "*" refers to estimated values.

S10 Photovoltaic effects of ITO/PEDOT:PSS/WSe_2/ZnO/Al

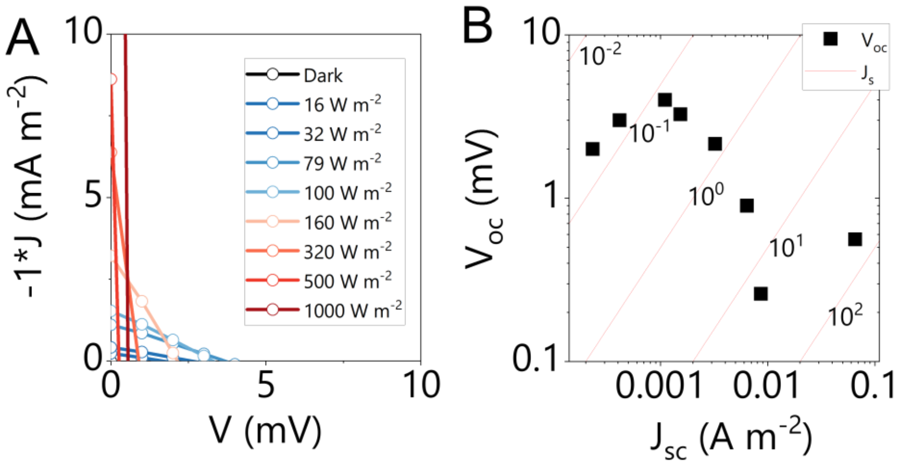

Fig. S8. (A) *J*-*V* curve of ITO/PEDOT:PSS/WSe_2/ZnO/Al under various *F*, (B) The plot of the extracted V_{oc} versus J_{sc}.

The photovoltaic effect can be found around origin of the *J*-*V* curves (Fig. S8A). J_{sc} increases with *F* while V_{oc} only increases with *F* at *F* <100 W m^{-2}. This indicates that thermal effect due to higher light intensity would increase J_s and leads to smaller V_{oc}, which is widely observed in other type of solar cells.[16]

The extracted V_{oc} is plotted against J_{sc} in Fig. S8B. V_{oc} is described by the following equation.[17]

$$V_{oc} = \frac{nkT}{q}\ln(1+\frac{J_{sc}}{J_s}) \tag{2}$$

Taking *n*=2, several plots with various J_s values can be made in the figure. The increase of J_s with *F* can be clearly visualized, which is consistent with the fitting results in Figure 4B.

S11 Calculation of photo-current density J_{ph}

For simplicity, the obtained heterojunction can be modelled as a PN junction, where WSe_2 serves as a p-type material and ZnO serves as a n-type material.

The built-in potential Φ_b for the heterojunction can be estimated by the E_f difference between two semiconductors. As the E_f of WSe_2 is close to -5.1 eV (PEDOT:PSS) according to the experimental observations, we estimate an E_f value of around -5.1 eV. ZnO is a highly doped semiconductor and its E_f will be very close to its CBM. Taking E_f of -4.6 eV for ZnO, Φ_b should be around 0.5 eV.

The depletion width W for a PN junction is given by:

$$W_p = \sqrt{\frac{2\varepsilon_0\varepsilon_r}{q}\frac{\Phi_b N_d}{N_a(N_a+N_d)}} \tag{3}$$

$$W_n = \sqrt{\frac{2\varepsilon_0\varepsilon_r}{q}\frac{\Phi_b N_a}{N_d(N_a+N_d)}} \tag{4}$$

where W_p and W_n are the depletion width on p-side and n-side semiconductors, respectively. N_d and N_a refer to donor concentration in ZnO and acceptor concentration in WSe_2, respectively. ϵ_0 and ϵ_r=7.7[18] are vacuum and relative permittivity, respectively. As WSe_2 is not deliberately doped, the donor concentration in ZnO arising from its oxygen vacancies will be much larger than the acceptor concentration in WSe_2 ($N_d >> N_a$). This results in W_n to be very small. Moreover, $N_d/(N_a + N_d)$ will be approximately 1. Taking previously reported intrinsic carrier density as the acceptor concentration of WSe_2 that N_a~10^{13} cm^{-3},[19] we can find W_p to be around 6.53 µm. The depletion width is much larger than the thickness t of WSe_2 ~20 nm in this study. Thus, we can assume the WSe_2 layer is fully depleted.

J_{ph} at 0 V (J_{sc}) depends on the absorption of the material, diffusion length and the depletion width, which is described by equation.

$$J_{ph} = q\left[G_p(L_p+W_p)+G_n(L_n+W_n)\right] \tag{5}$$

G is the optical generation rate of the material. $L=\sqrt{D\tau}$ is the diffusion length of the minority carriers in the corresponding material, where $D=\frac{kT}{q}\mu$ is the diffusion coefficient and μ is mobility, and τ is the photo-carrier lifetime. Considering the small absorption coefficient of ZnO in visible regime (small G_n), the ZnO related terms can be therefore neglected and will not be used for further calculation. This allows equation (5) to be re-written as:

$$J_{ph}=qG_p(L_p+W_p) \tag{6}$$

The light intensity at the WSe_2/ZnO interface is $Fe^{-\alpha t}$ if the optical loss from ITO and PEDOT:PSS is ignored. Therefore, $G_{WSe_2}=\frac{\alpha F}{h\nu}e^{-\alpha t}$,where h is Planck's constant, v is the frequency of the light, and t is the film thickness. G_p can be averaged in visible regime (400-700 nm) to be 5.84×10^{28} m^{-3} s^{-1}. The carrier lifetime and mobility are unknown. However, even L_p is small, the sum of L_p and W_p should be at least larger than 20 nm (thickness of WSe_2). We can calculate the expected J_{ph} at 0 V should be at least 116.8 A m^{-2} at 1000 W m^{-2}.

The theoretical value of J_{ph} is order of magnitudes higher than the experimental value (6.5×10^{-2} A m^{-2}). This implies that either G_p or the sum of L_p and W_p to be small. As is known that the optical generation of TMDs is superior from above calculation, the reason can only be the latter. The device is limited by both short diffusion length and narrow depletion width on the WSe_2 side.

S12 Comparison of photo-responsivity

Table S2 Comparison of photo-responsivity from photodetectors based on LPE WSe_2.

No.	Device			Performance		Ref.
	Type	Structure	Method	Light source	R_{ph} ($A\ W^{-1}$)	
1	PHJ	Ag/WSe_2/p-Si/Ag	Drop cast	670 nm	1.15 at -1.5 V	[8]
2		Ag/WSe_2/n-Si/Ag	Drop cast	LED	8.61e-2 at 0 V	[20]
3		Ag/WSe_2/p-Si/Ag	Electrophoresis	520 nm	*8.4e-2 at -2 V	[21]
4		ITO/PEDOT:PSS/WSe_2/ZnO/Al	Spray coat	AM1.5D	6.08e-3 at -1 V<br>6.53e-5 at 0 V<br>1.5 at 1 V	Our work
5	IP	Ag/WSe_2/Ag	Dip coat	590 nm	1.78e-2 at 5 V	[22]
6	IP	Ag/WSe_2/Ag	Dip coat	670 nm	*6e-3 at 5 V	[23]
7	OoP	CNT/WSe_2/CNT	Aerosol-jet	660 nm	1 at 1 V	[24]
8	IP	Gra/WSe_2/Gra	Drop cast	670 nm	6.66e-3 at 1 V	[25]
9	IP	ITO/WSe_2/ITO	Drop cast	Visible light	3.65e-6 at 1 V	[26]
10	IP	ITO/Cu:WSe_2/ITO	Drop cast	670 nm	9.31e-5 at 0 V	[27]
11	IP	Ag/WSe_2:PVC/Ag	-	470 nm	3.31e-4 at 20 V	[28]

Note: Device type: “PHJ”, “IP” and “OoP” refer to planar heterojunction, in-plane and out-of-plane Ohmic-contacted devices, respectively. The symbol “*” refers to estimated values.

Reference

1. E. Frau, Y. Zhang, L. Viau, F. Jurin, C. Buron, C. Filiatre, S. Schintke, **2020**, 1.

2. S. K. Lee, D. Chu, D. Y. Song, S. W. Pak, E. K. Kim, *Nanotechnology* **2017,** *28*, 195703.

3. S. K. Lee, D. Chu, J. Yoo, E. K. Kim, *Sol Energ Mat Sol C* **2018,** *184*, 9.

4. M. Patel, P. M. Pataniya, V. Patel, C. K. Sumesh, D. J. Late, *Sol. Energy* **2020,** *206*, 974.

5. S. Mukherjee, S. Biswas, S. Das, S. K. Ray, *Nanotechnology* **2017,** *28*, 135203.

6. M. Patel, P. Pataniya, H. Vala, C. K. Sumesh, *J. Phys. Chem. C* . **2019,** *123*, 21941.

7. S. Jana, S. Ray, S. Mukherjee, *ACS Appl. Nano Mater.* **2021,** *4*, 1877.

8. S. Kapatel, C. K. Sumesh, *Opt. Mater.* **2022,** *129*.

9. M. Patel, P. M. Pataniya, C. K. Sumesh, *Mater. Res. Bull.* **2022,** *145*.

10. D. Thangaraju, R. Marnadu, V. Santhana, A. Durairajan, P. Kathirvel, J. Chandrasekaran, S. Jayakumar, M. Valente, D. C. J. C. Greenidge, **2020,** *22*, 525.

11. J. Ye, X. Li, J. Zhao, X. Mei, Q. Li, *Nanoscale Res. Lett.* **2015,** *10*, 454.

12. J. Kim, D. Rhee, O. Song, M. Kim, Y. H. Kwon, D. U. Lim, I. S. Kim, V. Mazánek, L. Valdman, Z. Sofer, J. H. Cho, J. Kang, *Adv. Mater.* **2022,** *34*, 2106110.

13. B. Parida, S. Kim, M. Oh, S. Jung, M. Baek, J.-H. Ryou, H. Kim, *Materials Science in Semiconductor Processing* **2017,** *71*, 29.

14. K. H. Modi, P. M. Pataniya, V. Patel, C. K. Sumesh, *Solar Energy* **2021,** *221*, 412.

15. A. Umar, M. S. Akhtar, R. I. Badran, M. Abaker, S. H. Kim, A. Al-Hajry, S. Baskoutas, *Appl. Phys. Lett.* **2013,** *103*.

16. a) S. Chander, A. Purohit, A. Sharma, Arvind, S. P. Nehra, M. S. Dhaka, *Energy Reports* **2015,** *1*, 104; b) X. Cai, S. Zeng, X. Li, J. Zhang, S. Lin, A. Lin, B. Zhang, Effect of light intensity and temperature on the performance of GaN-based p-i-n solar cells. In *2011 International Conference on Electrical and Control Engineering*, **2011**; pp 1535.

17. S. Fonash, *solar cell device physics*. Elsevier: **2012**.

18. A. Laturia, M. L. Van de Put, W. G. Vandenberghe, *npj 2D Materials and Applications* **2018,** *2*.
19. A. G. Kelly, T. Hallam, C. Backes, A. Harvey, A. S. Esmaeily, I. Godwin, J. Coelho, V. Nicolosi, J. Lauth, A. Kulkarni, S. Kinge, L. D. Siebbeles, G. S. Duesberg, J. N. Coleman, *Science* **2017,** *356*, 69.
20. P. M. Pataniya, C. K. Zankat, M. Tannarana, A. Patel, S. Narayan, G. K. Solanki, K. D. Patel, P. K. Jha, V. M. Pathak, *Materials Research Bulletin* **2019,** *120*, 110602.
21. A. B. Patel, P. Chauhan, K. Patel, C. K. Sumesh, S. Narayan, K. D. Patel, G. K. Solanki, V. M. Pathak, P. K. Jha, V. Patel, *ACS Sustainable Chemistry & Engineering* **2020,** *8*, 4809.
22. P. Pataniya, C. K. Zankat, M. Tannarana, C. K. Sumesh, S. Narayan, G. K. Solanki, K. D. Patel, V. M. Pathak, P. K. Jha, *ACS Applied Nano Materials* **2019,** *2*, 2758.
23. D. Kannichankandy, P. M. Pataniya, C. K. Zankat, M. Tannarana, V. M. Pathak, G. K. Solanki, K. D. Patel, *Applied Surface Science* **2020,** *524*.
24. Y. Li, X. Feng, M. Sivan, J. F. Leong, B. Tang, X. Wang, J. N. Tey, J. Wei, K. W. Ang, A. V. Y. Thean, *IEEE Sensors Journal* **2020,** *20*, 4653.
25. P. M. Pataniya, C. K. Sumesh, *Synthetic Metals* **2020,** *265*.
26. B. L. Chauhan, S. A. Bhakhar, P. M. Pataniya, S. U. Gupta, G. K. Solanki, V. M. Pathak, V. Patel, *J. Mater. Sci.: Mater. Electron.* **2022,** *33*, 10314.
27. B. L. Chauhan, S. A. Bhakhar, P. M. Pataniya, G. K. Solanki, V. M. Pathak, *Opt. Mater.* **2022,** *133*.
28. D. Kannichankandy, P. M. Pataniya, V. Dhamecha, V. M. Pathak, G. K. Solanki, *Current Applied Physics* **2022,** *39*, 140.
